# Piperine, a black pepper compound, induces autophagy and cellular senescence mediated by NF-κB and IL-6 in acute leukemia

**DOI:** 10.1186/s12906-024-04641-9

**Published:** 2024-09-28

**Authors:** Kantorn Charoensedtasin, Wasinee Kheansaard, Sittiruk Roytrakul, Dalina Tanyong

**Affiliations:** 1 Department of Clinical Microscopy, Faculty of Medical Technology, Mahidol University, 999 Phuttamonthon sai 4 Road, Salaya, Phuttamonthon, Nakhon Pathom, 73170 Thailand; 2 Functional Proteomics Technology Laboratory, Functional Ingredients and Food Innovation Research Group, National Center for Genetic Engineering and Biotechnology, National Science and Technology for Development Agency, Pathum Thani, 12120 Thailand

**Keywords:** Leukemia, Piperine, Autophagy, Senescence

## Abstract

Acute leukemia is characterized by abnormal white blood cell proliferation with rapid onset and severe complications. Natural compounds, which are alternative treatments, are widely used in cancer treatment. Piperine, an alkaloid compound from black pepper, exerts anticancer effects through the cell death signaling pathway. Autophagy and senescence signaling pathways are considered target signaling pathways for cancer treatment. In this study, we investigated the effects of piperine via autophagy and senescence signaling pathways in NB4 and MOLT-4 cells. The MTT assay results demonstrated that piperine significantly decreased the viability of NB4 and MOLT-4 cells. Piperine induced autophagy by increasing LC3, Beclin-1 and ULK1 and decreasing mTOR and NF-κB1 expression in NB4 and MOLT-4 cells. In addition, piperine increased senescence-associated beta-galactosidase fluorescence intensity by increasing p21 and IL-6 expression while decreasing CDK2 expression in NB4 and MOLT-4 cells. In conclusion, our study provides additional information about the induction of autophagy and senescence by piperine in acute leukemia.

**Supplementary Information:**

The online version contains supplementary material available at 10.1186/s12906-024-04641-9.

## Background

Acute leukemia is a hematological malignancy characterized by defects in the proliferation of hematopoietic stem cells. It is characterized by rapid onset and severe complications such disseminated intravascular coagulation (DIC). In 2022, the incidence of leukemia was approximately 487,000 new cases and 305,000 deaths worldwide [1]. There is still an increasing trend of leukemia incidence in Thailand. The risk factors leading to leukemia include smoking, exposure to certain chemicals, radiation exposure or genetic defects [2]. Although there are various treatments for leukemia, such as chemotherapy, there are side effects, including nausea, vomiting or disease relapse. Anticancer compounds derived from natural sources, which are alternative treatments, provide better outcomes in leukemia treatment. Additionally, most natural compounds are less toxic to normal cells [3].

Piperine, a major alkaloid compound in *Piper nigrum* or black pepper, is widely utilized in multiple applications worldwide, such as traditional medicine. Piperine exhibits various pharmacological properties, including antioxidant, anti-inflammatory and antibacterial effects [4, 5]. Moreover, piperine has been reported to have effective anticancer activity in various cancer types [6, 7].

Autophagy and senescence signaling pathways are involved in cell death pathways to eliminate cancer. A target autophagy and senescence protein of piperine could be useful for monitoring the response to acute leukemia treatment [8]. Additionally, autophagy and the senescence signaling pathway in cancer cells are potential targets of natural compounds [9, 10]. Autophagy is a type II programmed cell death process involving the self-degradation of cellular components initiated by stress conditions, e.g., starvation and oxygen deprivation. Autophagy is divided into initiation, elongation, maturation, fusion, and degradation. In brief, organelles are engulfed to form autophagosomes, which then fuse with lysosomes to degrade cellular components [11–13]. Cellular senescence is an irreversible cell cycle arrest in which metabolic processes in cells are initiated by persistent DNA damage or chromosomal telomere shortening from different inducers. In most previous works, cellular senescence in cancer cells was induced by chemotherapeutic drugs, called therapy-induced senescence (TIS) [14, 15]. Many studies have demonstrated that autophagy and senescence are induced by various natural compounds, resulting in cancer cell death via different target proteins [9, 16–18].

Therefore, this project aimed to study the effect of piperine on autophagy and senescence signaling pathways via target proteins of piperine in acute leukemia.

## Methods

### Chemicals and reagents

**Fig. 1:**
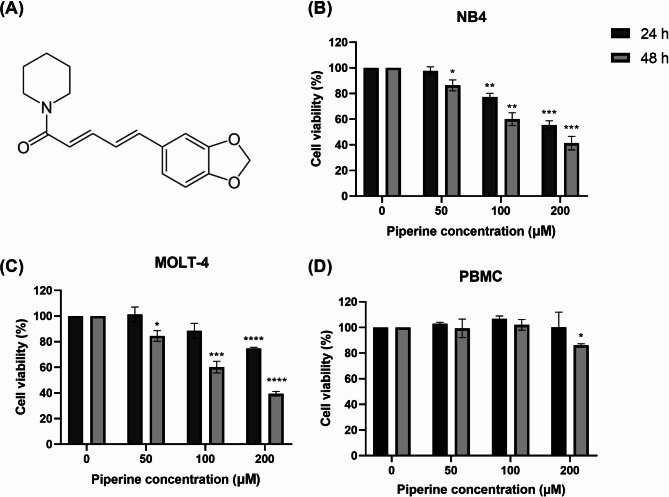
Inhibitory effects of piperine on NB4 and MOLT-4 cells. (**A**) The structure of piperine. NB4 cells, MOLT-4 cells and PBMCs were treated with 0, 50, 100 or 200 μM piperine for 24 h. and 48 h. The percentage of cell viability was analyzed using an MTT assay. Inhibitory effects of piperine on (**B**) NB4 cells, (**C**) MOLT-4 cells and (**D**) PBMCs at 24 h. and 48 h. The data are expressed as the mean ± S.E.M. of three independent experiments. *p < 0.05, **p < 0.01, ***p < 0.001 and ****p < 0.0001 indicate statistically significant differences compared with the control group

All chemicals and reagents used in this research are listed in Supplementary Table S1. The purity of the piperine was greater than 97%. The structure of piperine is shown in Fig. 1A.

### Leukemia cell culture and PBMC isolation

The human acute promyelocytic leukemia cell line (NB4) and the human acute lymphocytic leukemia cell line (MOLT-4) were purchased from Cell Lines Service (Eppelheim, Germany). NB4 and MOLT-4 cells were cultured in RPMI-1640 media supplemented with 10% (v/v) fetal bovine serum (FBS) and 1% (v/v) penicillin‒streptomycin. NB4 and MOLT-4 cells were incubated in a humidified incubator with 5% CO_2_ at 37 °C.

Peripheral blood mononuclear cell (PBMC) isolation was performed using the Ficoll‒Hypaque method (Ethics approval no. MU-CIRB 2023/125.1204 by the Mahidol University Central Institutional Review Board). Briefly, whole blood from healthy participants was mixed with PBS at a ratio of 1:1 and then gently overlaid on Lymphoprep™ solution. After that, whole blood was separated, and PBMCs in the intermediate phase of blood collection were collected. PBMCs were counted using trypan blue and then seeded into 96-well plates for MTT experiments.

### Determination of cell viability by MTT assay

NB4 cells, MOLT-4 cells and PBMCs were treated with various concentrations of piperine (0, 50, 100, or 200 μM) for 24 h. and 48 h. in a 96-well plate. Then, 5 mg/ml MTT solution was added to each well, and the plates were incubated for 4 h. The formazan crystals were dissolved in 10% SDS in 0.01 M HCl, followed by incubation overnight to develop color. The formazan absorbance was measured using a microplate reader at a wavelength of 570 nm (BioTek Instruments, Inc., Winooski, VT, USA). The half maximal inhibitory concentration (IC_50_) was calculated from equation of various concentration of piperine and used in further experiments.

### Prediction of target proteins associated with autophagy and senescence signaling pathways by bioinformatic analysis

The target proteins of piperine were retrieved from the computational tool STITCH database (http://stitch.embl.de/) using the keyword “Piperine” and specifying the species as *Homo sapiens*. The minimal required interaction score was set to high confidence (0.700), and the maximum number of interactors to display was limited to 40. Protein‒protein interactions between target proteins of piperine and representative proteins involved in autophagy and senescence, including mTOR, ULK1, p21 and CDK2, were assessed using STITCH (http://stitch.embl.de/). The target proteins that interacted with at least 2 representative proteins were chosen as candidate proteins. The candidate proteins with the highest interaction scores that are associated with autophagy and senescence were chosen for further experiments.

### LC3 level determination by flow cytometry

NB4 and MOLT-4 cells were treated with the IC_50_ piperine (150 μM) for 48 h. To assess autophagy activity, LC3 levels were determined using FlowCellect™ Autophagy LC3 Antibody-based Assay Kits (EMD Milipore Corporation, Germany) following the manufacturer’s protocol. Briefly, 10 μl of autophagy reagent A was added to piperine-treated NB4 and MOLT-4 cells to stop the cellular degradation process. NB4 and MOLT-4 cells were harvested and washed with PBS. 100 μl autophagy reagent B was then added to permeabilized NB4 and MOLT-4 cells before they were stained with FITC-conjugated anti-LC3 for 15 min at room temperature in the dark place. The LC3 mean fluorescence intensity (MFI) was analyzed using a FACSCantoII flow cytometer. The LC3 MFI value and histogram were created using BD FACSDiva™ Software.

### Determination of the level of senescence-associated β-galactosidase (SA-β-gal) by flow cytometry

NB4 and MOLT-4 cells were treated with the IC_50_ piperine (150 μM) for 48 h. To assess SA-β-gal levels, a Cell Event™Senescence Green Flow Cytometry Assay Kit (Invitrogen, USA) was used following the manufacturer’s protocol. Briefly, NB4 and MOLT-4 cells were harvested and washed with PBS. NB4 and MOLT-4 cells were subsequently fixed with 2% paraformaldehyde in PBS for 10 min. After that, the NB4 and MOLT-4 cells were stained with a Cell Event™ Senescence Green Probe and then incubated for 90 min at 37 °C in an incubator without carbon dioxide. The mean fluorescence intensity of SA-β-Gal in NB4 and MOLT-4 cells was determined using a FACSCanto II flow cytometer. The SA-β-gal MFI value and histogram were created using BD FACSDiva™ Software.

### Gene expression determination by real-time quantitative polymerase chain reaction (RT‒qPCR)

NB4 and MOLT-4 cells were treated with the IC_50_ piperine (150 μM) for 48 h. NB4 and MOLT-4 cells were subsequentlyharvested and washed twice with PBS. RNA extraction was performed using GENEzol™ reagent (New England Biolab, Inc., Ipswich, MA, USA) following the manufacturer’s instructions. The RNA concentration was determined using a Nanodrop2000 spectrophotometer (Thermo Scientific, Waltham, MA, USA). Then, 500 μg/ml–1000 μg/ml of extracted RNA was converted to cDNA by using a RevertAid First Strand cDNA Synthesis Kit (Thermo Scientific, Waltham, MA, USA). qPCR was performed using Luna real-time PCR master mix (New England Biolab, Inc., Ipswich, MA, USA) on a Bio-Rad CFX96 touch™ real-time PCR system. The mRNA expression level was normalized to that of 18 S ribosomal RNA (rRNA). Relative mRNA expression was calculated using the 2^−ΔΔCT^ method. The sequences of the primers used in this study are shown in Table 1.

**Table 1:** Sequences of the primers used in this study

Gene	Sequence (5’ → 3’)
mTOR	F: GGAGAAATTTGATCAGATACCCAGT R: CCACAGAAAGTAGCCCCAGG
BECN1	F: GAGTTTCAAGATCCTGGACCGTGTCA R: CTGTTGGCACTTTCTGTGGACATCA
ULK1	F: GGCAAGTTCGAGTTCTCCCG R: CGACCTCCAAATCGTGCTTCT
P21	F: GTCAGTTCCTTGTGGAGCCG R: CCATTAGCGCATCACAGTCG
CDK2	F: TGGATGCCTCTGCTCTCACTG R: GAGGACCCGATGAGAATGGC
NF-κB1	F: CTTAGGAGGGAGAGCCCAC R: GCAGTGCCATCTGTGGTTGA
IL-6	F: TGAACTCCTTCTCCACAAGCG R: GAAGGCAGCAGCAGGCAACAC
18 s ribosomal RNA	F: ATTAAGGGTGTGGGCCGAAG R: CAGGTCTTCACGGAGCTTGT

### Protein determination by western blotting

NB4 and MOLT-4 cells were treated with the IC_50_ piperine (150 μM) for 48 h. NB4 and MOLT-4 cells were subsequently harvested and washed twice with PBS. Protein cell lysates were collected using RIPA lysis buffer supplemented with protease inhibitor. The protein concentration of the cell lysate was determined using a Dual-Range BCA Protein Assay Kit (Visual Protein, Taipei, Taiwan). Protein cell lysates were separated by SDS‒PAGE. The proteins subjected to SDS‒PAGE were subsequently transferred to polyvinylidene fluoride (PVDF) membranes. After that, the PVDF membrane was blocked with EveryBlot Blocking Buffer (Bio-Rad, Inc., Hercules, CA, USA) or 5% nonfat milk at room temperature. Primary antibodies against mTOR, CDK2, ULK1, p21 (Cell Signaling Technology, Danvers, MA, USA), Beclin-1, α-tubulin (Proteintech, USA) and NF-κB1 (Invitrogen, USA) were incubated with the PVDF membranes overnight at 4 °C. Next, the membranes were incubated with horseradish peroxidase (HRP)-conjugated secondary antibodies (Cell Signaling Technology, Danvers, MA, USA). Protein expression on the PVDF membrane was observed using enhanced chemiluminescence (ECL) substrate and was determined using Lab™ software (Bio-Rad, Inc., Hercules, CA, USA). Relative protein expression was normalized to that of α-tubulin.

### IL-6 secretion determination by ELISA

NB4 and MOLT-4 cells were treated with the IC_50_ piperine (150 μM) for 48 h. The supernatant was harvested and centrifuged at 1,500 rpm for 5 min to remove cells. ELISA was performed following the manufacturer’s instructions (ELK Biotechnology, USA). IL-6 protein level was determined using Human IL6(Interleukin 6) ELISA kit (ELK Biotechnology, USA). First, 100 μl of cell culture supernatant was added to the appropriate well and then incubated for 80 min at 37 °C. After that, supernatant was poured out and the wells were washed with wash solution. Then, 100 μl of biotinylated antibody solution was added to each well and incubated for 50 min at 37 °C. The biotinylated antibody mixture was poured out and the wells were washed with wash solution. After that, 100 μl of streptavidin-HRP solution was added to each well and then incubated for 50 min at 37 °C, after which the mixture was poured out and washed with wash solution. 50 μl of TMB substrate solution was added to each well and then incubated for 20 min at 37 °C in the dark. After that, 50 μl of Stop reagent was added to each well. The absorbances were immediately determined using a microplate reader at a wavelength of 450 nm (BioTek Instruments, Inc., Winooski, VT, USA).

### Statistical analysis

All data throughout this study are presented as the mean ± standard error of the mean (S.E.M.). The graphs were created using GraphPad Prism version 8.0 (GraphPad Inc., San Diego, CA, USA). Comparative statistical analysis was performed using GraphPad Prism version 8.0 (GraphPad Inc., San Diego, CA, USA). Unpaired Student’s t tests were performed to compare two groups. A *p* value < 0.05 was considered to indicate a significant difference.

## Results

### Piperine decreases the percentage of NB4 and MOLT-4 cell viability in a dose-dependent manner

NB4 and MOLT-4 cells were treated with piperine for 24 h. and 48 h. The percentage of cell viability was determined using the MTT assay. The structure of piperine is illustrated as an alkaloid compound containing nitrogen atoms located in a cyclic ring structure, as shown in Fig. 1A. As shown in Fig. 1B-D, piperine significantly decreased the percentage of viable NB4 and MOLT-4 cells in a dose-dependent manner for 24 h. and 48 h. In contrast, only 200 μM piperine was used for 48 h. exhibited cytotoxicity to PBMCs. In NB4 cells, the IC_50_ values of piperine were 224 μM ± 3.2 and 145 μM ± 5.3 at 24 h. and 48 h., respectively, whereas in MOLT-4, the IC_50_ values of piperine were 384 μM ± 4.2 and 156 ± 3.5 μM at 24 h. and 48 h., respectively. Therefore, piperine has a potential inhibitory effect on NB4 and MOLT-4 cells and is less cytotoxic to PBMCs.

### NF-κB1 and IL-6 are target proteins associated with autophagy and senescence

**Fig. 2:**
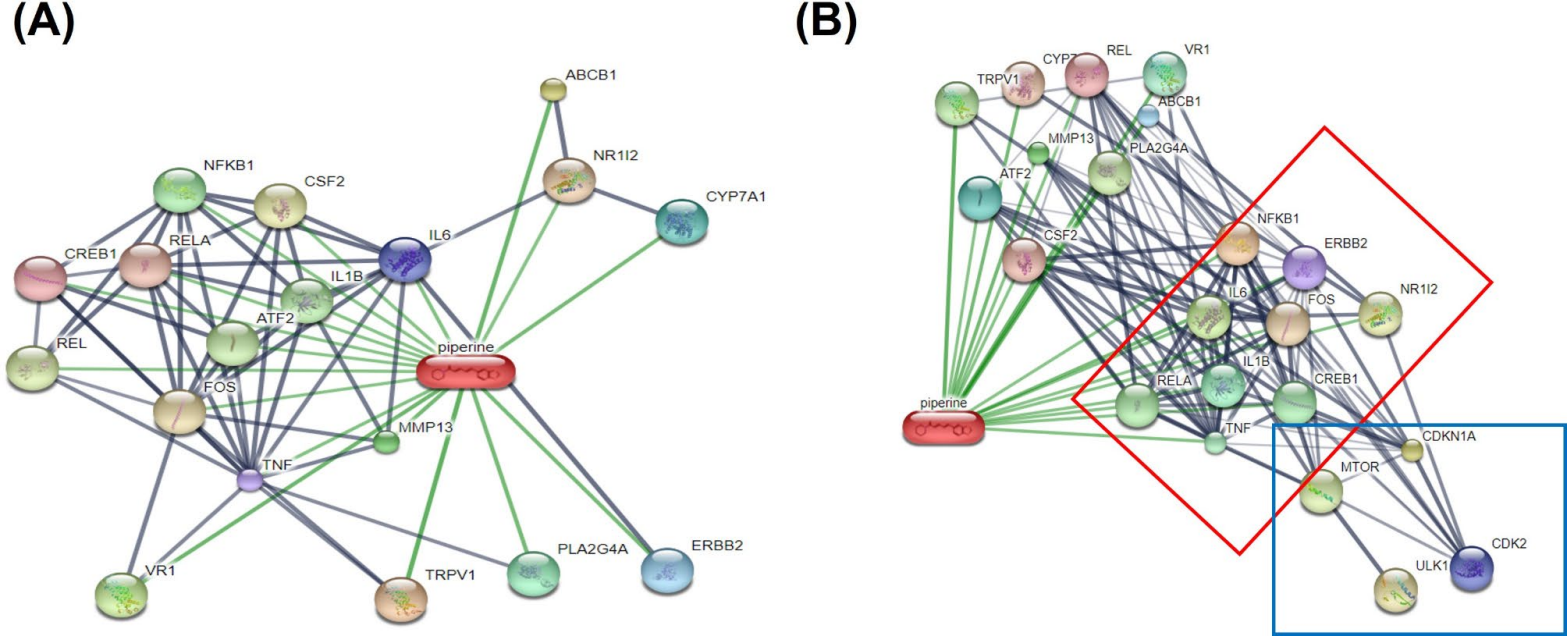
NF-κB1 and IL-6 are piperine-responsive target proteins involved in autophagy and senescence. (**A**) All possible piperine-responsive target proteins identified by the STITCH database. (**B**) Protein‒protein interactions of piperine-responsive target proteins and autophagy/senescence proteins, including mTOR, ULK1, CDKN1A (p21) and CDK2. The green lines represent proteins that directly interact with piperine. The red box indicates a group of proteins that interact with mTOR, ULK1, CDKN1A (p21) or CDK2. The blue box represents a group of autophagy and senescence proteins

To discover the target proteins of piperine, STITCH was utilized to generate possible target proteins of piperine. As shown in Fig. 2A, all the target proteins of piperine contained 18 proteins with interaction scores greater than 0.700. The interaction scores of the piperine-responsive target proteins are shown in Table 2. Next, mTOR, ULK1, p21 and CDK2, which are representative autophagic proteins, were added along with piperine-responsive target proteins. A protein‒protein interaction network was constructed using STITCH. ERBB2, IL6, IL1B, RELA (p65), TNF, CREB1, NFKB1, NR1l2 and FOS are piperine-responsive target proteins that interact with autophagy and senescence proteins, as shown in Fig. 2B. For the interaction scores between the target proteins and mTOR, the interaction scores of ERBB2, IL-6, RELA, TNF, CREB1, NF-κB1 and FOS were 0.769, 0.912, 0.938, 0.578, 0.604, 0.971 and 0.538, respectively. There was no target protein that directly interacted with ULK1. For CDKN1A (p21), the interaction scores of ERBB2, IL6, IL1B, RELA, TNF, CREB1, NFKB1, and FOS were 0.914, 0.903, 0.834, 0.472, 0.459, 0.952, 0.585, and 0.659, respectively. For CDK2, the interaction scores of ERBB2, IL6, RELA, CREB1, NFKB1, and FOS were 0.716, 0.472, 0.708, 0.675, 0.708 and 0.767, respectively. In summary, NFKB1 and IL-6 were selected for further investigation.

**Table 2:** List of piperine-responsive proteins

Proteins name	Proteins abbreviation	Interaction score
Transient receptor potential cation channel, subfamily V, member 1	TRPV1	0.918
ATP-binding cassette, subfamily B	ABCB1	0.859
Transient receptor potential cation channel subfamily V member 1	VR1	0.848
Matrix metallopeptidase 13 (collagenase 3)	MMP13	0.815
Phospholipase A2	PLA2G4A	0.8
Cytochrome P450, family 7, subfamily A, polypeptide 1	CYP7A1	0.8
Erythroblastic leukemia viral oncogene homolog	ERBB2	0.8
Interleukin 6	IL6	0.725
Tumor necrosis factor	TNF	0.725
cAMP responsive element binding protein 1	CREB1	0.7
Reticuloendotheliosis viral oncogene homolog A	RELA	0.7
Nuclear receptor subfamily 1, group I, member 2	NR1I2	0.7
FBJ murine osteosarcoma viral oncogene homolog	FOS	0.7
Colony stimulating factor 2	CSF2	0.7
v-rel reticuloendotheliosis viral oncogene homolog	REL	0.7
Activating transcription factor 2	ATF2	0.7
Interleukin 1, beta	IL1B	0.7
Nuclear factor of kappa light polypeptide gene enhancer in B cells 1	NFKB1	0.7

### Piperine induces autophagy in NB4 and MOLT-4 cells by regulating NF-κB1

**Fig. 3:**
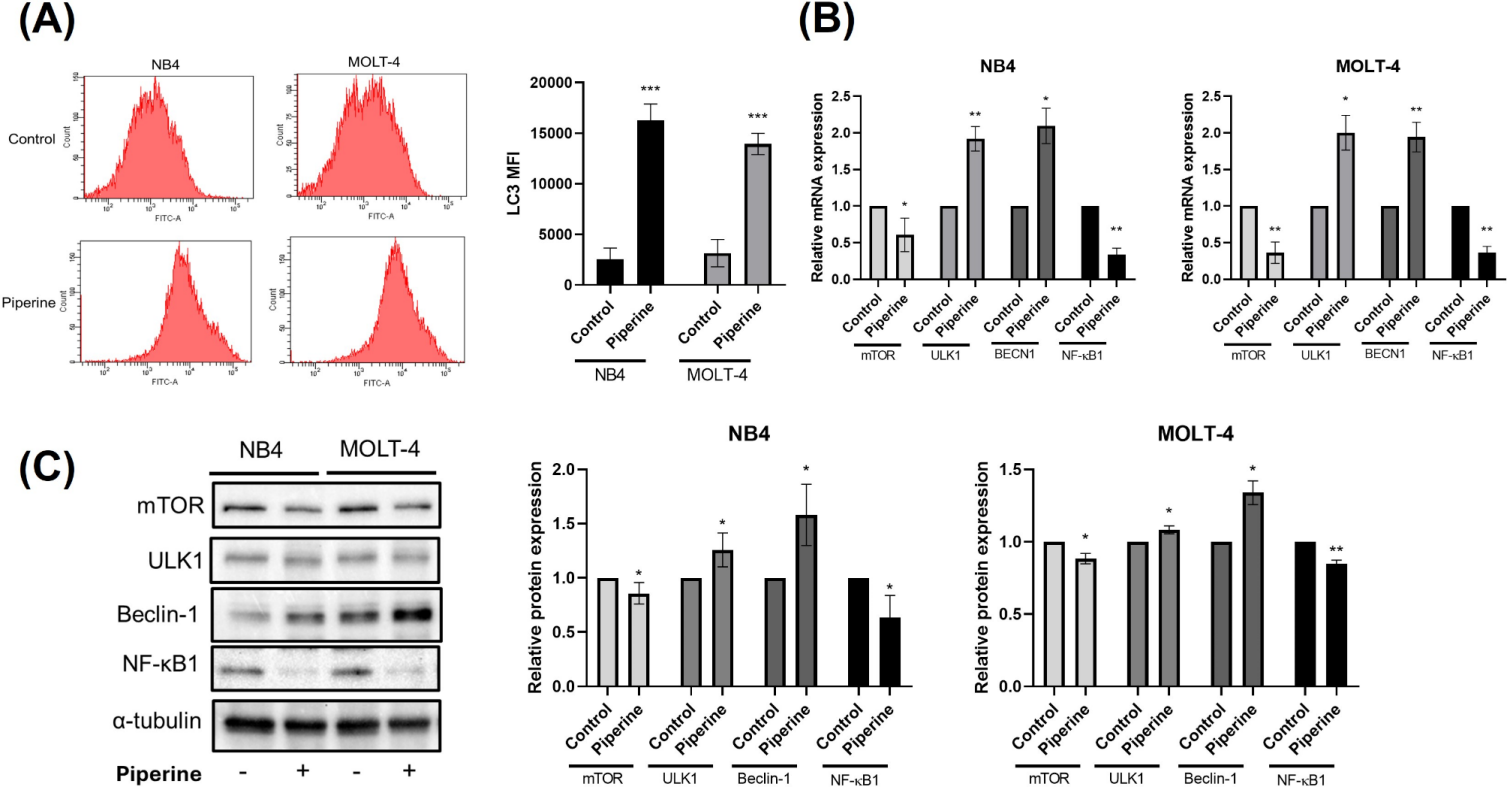
Induction of autophagy by piperine is mediated by NF-κB1 in NB4 and MOLT-4 cells. NB4 and MOLT-4 cells were treated with piperine at the IC_50_ for 48 h. LC3 expression in piperine-treated NB4 and MOLT-4 cells was analyzed using flow cytometry. mTOR, ULK1 and NF-κB1 expression was determined using RT‒qPCR and western blotting. (**A**) Histogram of LC3-conjugated FITC fluorescence and the mean fluorescence intensity (MFI) of LC3 in piperine-treated NB4 and MOLT-4 cells. (**B**) mTOR, ULK1, BECN1 and NF-κB1 gene expression in piperine-treated NB4 and MOLT-4 cells. (**C**) Western blot showing the protein levels of mTOR, ULK1, Beclin-1 and NF-κB1 and the levels of the mTOR, ULK1, Beclin-1 and NF-κB1 proteins in piperine-treated NB4 and MOLT-4 cells. The data are expressed as the mean ± S.E.M. of three independent experiments. *p < 0.05, **p < 0.01 and ***p < 0.001 indicate statistically significant differences compared withthe control group

To assess autophagy-related cell death in piperine-treated NB4 and MOLT-4 cells, LC3 levels were determined using flow cytometry. Compared with control, piperine significantly increased the mean fluorescence intensity of LC3 in NB4 and MOLT-4 cells, as shown in Fig. 3A. Next, the expression of autophagic genes and proteins, including mTOR and ULK1, was investigated by RT‒qPCR and western blotting, respectively. Compared with the control, piperine significantly induced ULK1 and BECN1 gene expression in NB4 and MOLT-4 cells but significantly reduced mTOR expression in NB4 and MOLT-4 cells. NF-κB1, an NF-κB family protein, was significantly lower in NB4 and MOLT-4 cells after piperine treatment than in control cells, as shown in Fig. 3B. Similarly, compared with the control, piperine significantly increased ULK1 and Beclin-1 protein expression but significantly decreased mTOR and NF-κB1 protein expression in NB4 and MOLT-4 cells, as shown in Fig. 3C. These findings suggest that piperine can suppress NF-κB1, which is a piperine-responsive target protein; consequently, autophagy is induced via mTOR, ULK1 and Beclin-1 in NB4 and MOLT-4 cells.

### Piperine induces senescence in NB4 and MOLT-4 cells through p21 and CDK2 and increases the senescence-associated secretory phenotype of IL-6 in these cells

**Fig. 4:**
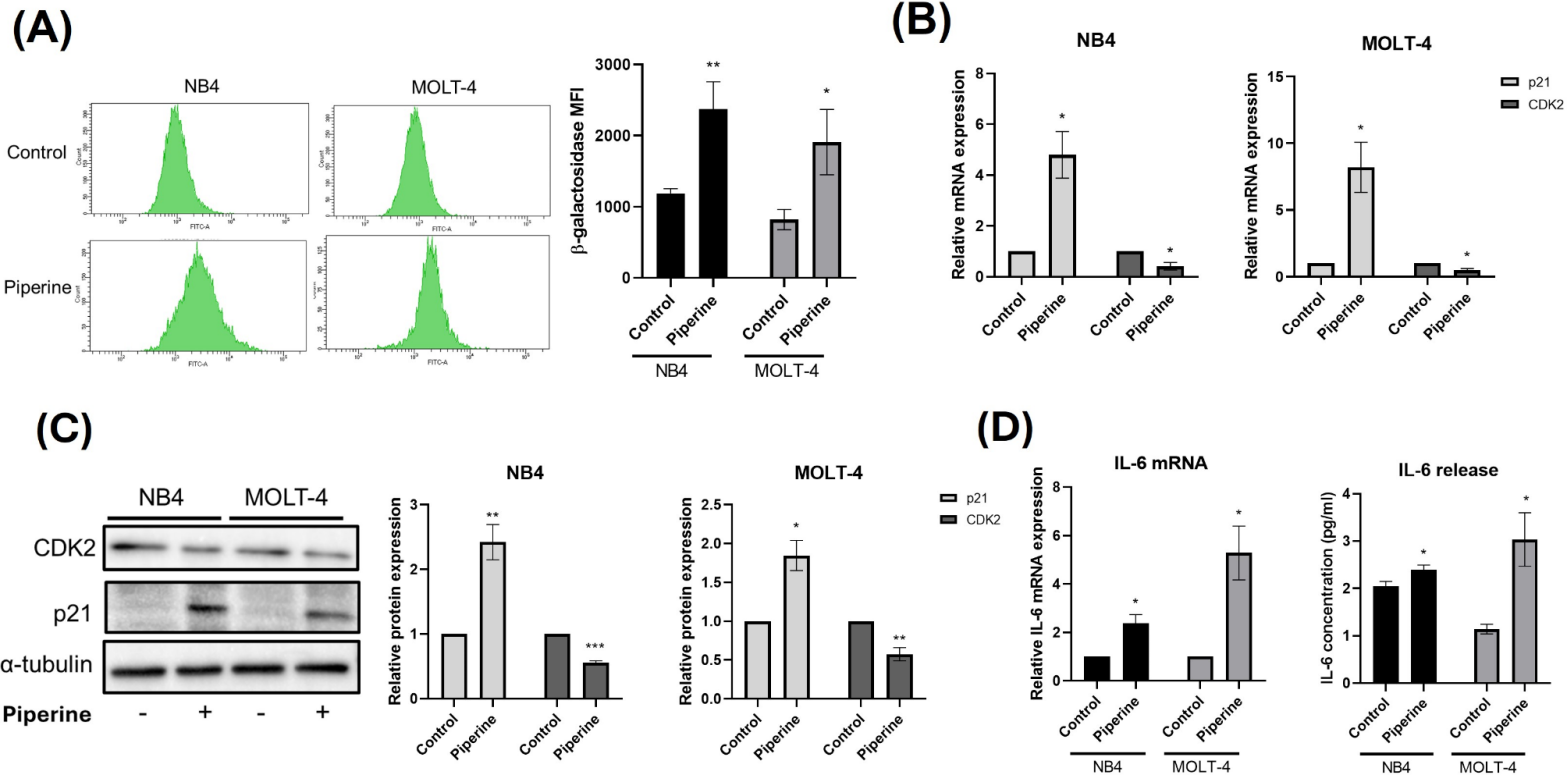
Induction of senescence by piperine mediated by IL-6 in NB4 and MOLT-4 cells. NB4 and MOLT-4 cells were treated with piperine at the IC_50_ for 48 h. The levels of senescence-associated β-galactosidase (SA-β-gal) in NB4 and MOLT-4 cells were analyzed using flow cytometry. p21 and CDK2 expression was determined using RT‒qPCR and western blotting. The level of IL-6 was determined using RT‒qPCR and ELISA. (**A**) Histogram of SA-β-gal-conjugated FITC fluorescence and the SA-β-gal mean fluorescence intensity (MFI) in piperine-treated NB4 and MOLT-4 cells. (**B**) p21 and CDK2 gene expression in piperine-treated NB4 and MOLT-4 cells. (**C**) Western blot showing p21 and CDK2 protein bands and p21 and CDK2 protein expression in piperine-treated NB4 and MOLT-4 cells. (**D**) IL-6 gene expression and IL-6 release in piperine-treated NB4 and MOLT-4 cells. The data are expressed as the mean ± S.E.M. of three independent experiments. *p < 0.05, **p < 0.01 and ***p < 0.001 indicate statistically significant differences compared with the control group

To determine senescence activity in piperine-treated NB4 and MOLT-4 cells, senescence-associated β-galactosidase (SA-β-gal) was assessed using flow cytometry. As shown in Fig. 4A, compared with the control, piperine significantly increased the mean fluorescence intensity of SA-β-gal in NB4 and MOLT-4 cells. Compared with the control, piperine significantly increased p21 gene expression but significantly decreased CDK2 gene expression in NB4 and MOLT-4 cells, as depicted in Fig. 4B. Similarly, compared with the control, piperine significantly increased p21 expression but significantly decreased CDK2 expression in NB4 and MOLT-4 cells, as depicted in Fig. 4C. To determine the senescence-associated secretory phenotype (SASP), which is a characteristic of cellular senescence, IL-6 release was assessed using RT‒qPCR and ELISA. Compared with the control, piperine significantly increased IL-6 gene expression and significantly increased IL-6 release from NB4 and MOLT-4 cells as shown in Fig. 4D. These findings indicate that piperine can induce senescence mediated by p21, CDK2 and IL-6 in NB4 and MOLT-4 cells.

## Discussion

Natural compounds are complementary alternative treatments for various cancer cells because of their low cytotoxicity in normal cells and consequently fewer adverse effects in patients [19, 20]. In a recent study, we demonstrated the anticancer effect of piperine on NB4 and MOLT-4 leukemia cells via autophagy and senescence activation. According to previous reports, piperine has many pharmacological properties, such as anti-inflammatory, immunomodulatory, and antioxidant effects [4, 5]. Moreover, piperine has shown anticancer effects in many cancer types, such as colorectal cancer, cervical cancer, and hepatocellular carcinoma [21]. For example, piperine suppresses colorectal cancer cells via the Wnt/β-catenin pathway [22]. Similarly, piperine also inhibited K562 leukemia cells via the apoptosis signaling pathway [7]. In addition, piperine is often combined with other anticancer compounds to synergistically inhibit cancer cells and improve their pharmacokinetic properties. For example, the combination of apatinib and piperine inhibited HCT-116 colorectal cancer cells by regulating MDM-2 gene expression [23]. Similarly, Yapaset R. and Banjerdpongchai R. reported that the combination of gambodic acid and piperine synergistically suppressed cholangiocarcinoma cells through the apoptosis signaling pathway [24].

Autophagy is an intracellular self-degradation mechanism that responds to various conditions. It has dual functions, including cell survival and tumor suppression, depending on the stage of cancer development and environmental conditions [13, 25]. Most natural compounds are tumor suppressors. However, some compounds inhibit autophagy, which facilitates cancer development [26]. For example, cyclohexidimide inhibited starvation-induced autophagy by upregulating mTORC1 [27]. The results demonstrated that piperine induced the expression of LC3, ULK1 and Beclin-1 while reducing mTOR expression in NB4 and MOLT-4. In previous studies, piperine was shown to be associated with autophagy activation. For example, piperine activated autophagy by increasing LC3B protein levels in RWPE-1, LNCaP, DU145 and PC-3 prostate cancer cells [17]. One study reported the induction of autophagy by piperine in drug-resistant human leukemia cells [28]. Autophagy is initiated by inactivation of mammalian target of rapamycin complex 1 (mTORC1), leading to activation of the ULK1 complex. The ULK1 complex and class II phosphoinositide 3-kinase (PI3K)/BECN1 complex are required to initiate phagophore and organelle engulfment of double membranes. LC3 participates in autophagy signaling to facilitate the loading of cargoes or organelles into autophagosomes, which subsequently dock and fuse with lysosomes [29, 30]. These findings suggest that piperine can induce autophagy in acute leukemia.

In a recent study, bioinformatics analysis was used to assess protein‒protein interactions with autophagy proteins using STITCH. Protein‒protein interactions demonstrated NF-κB1, target protein of piperine interacts with mTOR. Our results demonstrated that piperine decreased NF-κB1 and mTOR expression, leading to increased autophagy in NB4 and MOLT-4 cells. Nuclear factor κB (NF-κB) is a transcription factor complex that controls the expression of many genes involved in cell survival, inflammation and drug resistance [31]. Many phytochemical compounds inhibit NF-κB activity, consequently reducing cancer progression via the deactivation of proliferation or activation of the apoptotic cell death pathway [32]. In addition, resveratrol, a natural polyphenolic phytochemical, increased the lysosomal permeability of autophagy through the inhibition of NF-κB expression in cervical cancer cells [33]. These findings suggest that NF-κB is associated with autophagy in cancer and that piperine can suppress NF-κB expression in leukemic cells. In contrast, persistent autophagy can negatively affect cancer cell survival, resulting in resistance to cell death [12, 34].

Cellular senescence is a state of cell cycle arrest in proliferating cells. According to the mechanism of cellular senescence, DNA damage and telomere shortening initially induce ataxia-telangiectasia mutated (ATM) and then phosphorylate p53 to consequently induce p21. This mechanism ultimately arrests the cell cycle by inhibiting CDK2, a kinase that promotes cell cycle progression, resulting in cellular senescence [15, 35]. The early stage of cellular senescence in cancer is thought to play a tumor suppressive role. Many natural compounds are considered to cause therapy-induced senescence (TIS) [9]. Our study demonstrated that piperine induced senescence-associated β-galactosidase (SA-β-gal) in NB4 and MOLT-4 cells. Many proteins interact with the senescence signaling pathway. In our study, protein‒protein interaction analysis by STITCH demonstrated that IL-6 interacted with p21 and CDK2. Additionally, piperine upregulated IL-6 leading to the induction of p21 and a reduction in CDK2 which is involved in the cell cycle arrest of NB4 and MOLT-4 cells.

. With respect to the effects of natural compounds on cancer senescence, punicalagin induces senescence in papillary thyroid carcinoma via the upregulation of p21 with increasing SASP [36]. The combination of piperine and pentagamavunon-1 (PGV-1) synergistically promotes the senescence of 4T1 breast cancer cells [37]. Similarly, piperine selectively induces cellular senescence by releasing SASP and IL-6 in HeLa cervical cancer cells while suppressing premature senescent human diploid fibroblasts (S-HDFs), similar to normal cells [38]. These findings support our findings of the induction of acute leukemia senescence by piperine.

In contrast, tumor cells in a persistent senescent state can release SASP factors, such as the proinflammatory cytokines IL-1α, IL-6 and IL-8, to mediate tumor progression in neighboring cells [39, 40]. Moreover, the SASP is activated by the binding of NF-κB to target genes [41]. However, our results demonstrated a decrease in NF-κB and an increase in IL-6 release. Thus, the impact of persistent senescence in cancer cells on SASP release caused by piperine treatment should be further investigated.

## Conclusion

According to our results, piperine can inhibit NB4 and MOLT-4 cell proliferation via autophagy and senescence signaling mediated by NF-κB1 and IL-6. We provide additional information on the inhibitory effect of piperine on acute leukemia via autophagy and senescence. Therefore, piperine could be a candidate treatment for acute leukemia in the future.

## Electronic supplementary material

Below is the link to the electronic supplementary material.

Supplementary Material 1

Supplementary Material 2

Supplementary Material 3

## Data Availability

The target proteins datasets of piperine during current study were obtained from STITCH database (http://stitch.embl.de/). The datasets used and analyzed during the current study are available from the corresponding author.

## Abbreviations

CDK2: Cyclin-dependent kinase 2
FITC: Fluorescein isothiocyanate
HCl: Hydrochloric acid
IC_50_: The half maximal inhibitory concentration
IL-6: Interleukin-6
MFI: Mean fluorescence intensity
mTOR: Mammalian target of rapamycin
MTT: (3-[4,5-dimethylthiazol-2-yl]-2,5 diphenyl tetrazolium bromide)
NF-κB1: Nuclear factor kappa-light chain enhancer of activated B cells 1
PBS: Phosphate-buffered saline
SA-β-gal: Senescence-associated beta-galactosidase
SASP: Senescence associated secretory phenotype
SDS: Sodium dodecyl sulfate
ULK1: Unc-51-like autophagy-activating kinase 1
