# Supplementary material for: Piperine, a black pepper compound, induces autophagy and cellular senescence mediated by NF-κB and IL-6 in acute leukemia
Source: BMC Complement Med Ther. 2024 Sep 28;24:343. doi: 10.1186/s12906-024-04641-9 (PMC11438257; doi:10.1186/s12906-024-04641-9)
Supplement: Supplementary file 1 — Supplementary Material 1 [file 12906_2024_4641_MOESM1_ESM.docx]

| **Supplementary table S1** Chemicals and reagents used in this paper | | |
| --- | --- | --- |
| **Chemical and Reagents** | **Catalog number** | **Brand** |
| Piperine | P49007-5G | Sigma‒Aldrich, USA |
| MTT | M6494 | Invitrogen, USA |
| RPMI-1640 | 1TFS-1CC-31800022 | Gibco™, USA |
| Fetal bovine serum | F7524 | Sigma‒Aldrich, USA |
| Penicillin‒streptomycin | SV30010 | HyClone™, Cytiva, USA |
| Lymphoprep™ solution | 04-03-9391/02 | STEMCELL Technologies, Canada |
| Dimethyl Sulfoxide for cell culture (DMSO) | 67-68-5 | PanReac Applichem, Spain |
| FlowCellect™ Autophagy LC3 Antibody-based Assay Kits | CS208214 | EMD Milipore Corporation, Germany |
| Cell Event™ Senescence Green Flow Cytometry Assay Kit | C10840 | Invitrogen, USA |
| GENEzol^TM^ reagent | HI15902 | Geneaid, Taiwan |
| RevertAid First Strand cDNA Synthesis Kit | K1622 | Thermo Scientific, Waltham, MA, USA |
| Luna real-time PCR master mix | M3003L | New England Biolab, Inc., Ipswich, MA, USA |
| mTOR | 2983 | Cell Signaling Technology, Danvers, MA, USA |
| CDK2 | 2546 | Cell Signaling Technology, Danvers, MA, USA |
| ULK1 | 8054 | Cell Signaling Technology, Danvers, MA, USA |
| P21 | 2947 | Cell Signaling Technology, Danvers, MA, USA |
| Beclin-1 | 66665-1-Ig | Proteintech, USA |
| α-tubulin | 11224-1-AP | Proteintech, USA |
| NF-κB1 | MA5-15870 | Invitrogen, USA |
| Anti-mouse IgG, HRP-linked Antibody | 7076 | Cell Signaling Technology, Danvers, MA, USA |
| Anti-rabbit IgG, HRP-linked Antibody | 7074 | Cell Signaling Technology, Danvers, MA, USA |
| Human IL6(Interleukin 6) ELISA kit | ELK1156 | ELK Biotechnology, USA |
