# Supplementary material for: Piperine, a black pepper compound, induces autophagy and cellular senescence mediated by NF-κB and IL-6 in acute leukemia
Source: BMC Complement Med Ther. 2024 Sep 28;24:343. doi: 10.1186/s12906-024-04641-9 (PMC11438257; doi:10.1186/s12906-024-04641-9)
Supplement: Supplementary file 2 — Supplementary Material 2 [file 12906_2024_4641_MOESM2_ESM.docx]

| **Piperine concentration (µM)** | **%Cell viability of NB4 (mean ± S.E.M)** | **p-value** | **%Cell viability of MOLT-4 (mean ± S.E.M)** | **p- value** | **%Cell viability of PBMC (mean ± S.E.M)** | **p- value** |
| --- | --- | --- | --- | --- | --- | --- |
| **24 h** | | | | | | |
| **0** | 100 ± 0 | - | 100 ± 0 | - | 100 ± 0 | - |
| **50** | 97.8 ± 3.1 | 0.5126 | 101.2 ± 5.8 | 0.8427 | 102.9 ± 0.7 | 0.9323 |
| **100** | 77.2 ± 3.0 | 0.0016* | 88.6 ± 5.7 | 0.1166 | 106.8 ± 1.3 | 0.5403 |
| **200** | 55.3 ± 3.4 | 0.0002* | 74.7 ± 0.9 | <0.0001* | 100.2 ± 6.781 | >0.9999 |
|  | **IC50 = 224 µM** | | **IC50 = 384 µM** | | - | |
| **48 h** | | | | | | |
| **0** | 100 ± 0 | - | 100 ± 0 | - | 100 ± 0 | - |
| **50** | 86.4 ± 4.3 | 0.0331* | 84.5 ± 4.1 | 0.0195* | 99.3 ± 4.2 | 0.9964 |
| **100** | 56.5 ± 6.3 | 0.0023* | 60.2 ± 4.6 | 0.0010* | 102.0 ± 2.4 | 0.9330 |
| **200** | 41.3 ± 5.3 | 0.0004* | 39.5 ± 1.8 | <0.0001* | 86.0 ± 0.7 | 0.0158* |
|  | **IC50 = 145 µM** | | **IC50 = 156 µM** | | - | |

**Supplementary table S2** Descriptive statistical results of percentage of cell viability
