# Supplementary material for: Piperine, a black pepper compound, induces autophagy and cellular senescence mediated by NF-κB and IL-6 in acute leukemia
Source: BMC Complement Med Ther. 2024 Sep 28;24:343. doi: 10.1186/s12906-024-04641-9 (PMC11438257; doi:10.1186/s12906-024-04641-9)

**Figure S1 Full uncropped blot of all proteins in the study.** (A) Full uncropped blots of Figure 3C including mTOR, ULK1, NF-κB1, Beclin-1 and α-tubulin proteins. (B) Full uncropped blots of Figure 4C including p21, CDK2 and α-tubulin proteins. Red squares were the proteins band used in the Figure results.

**(A)**

**
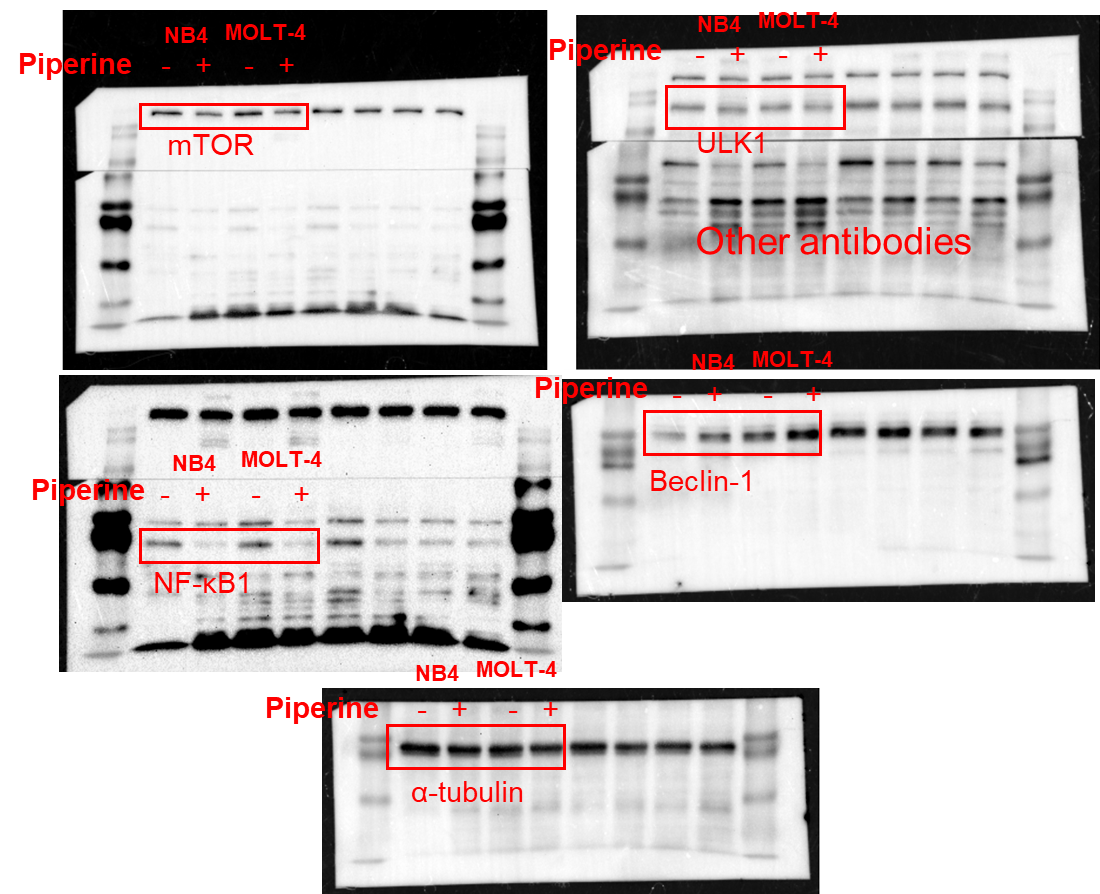
**

**(B)**

**
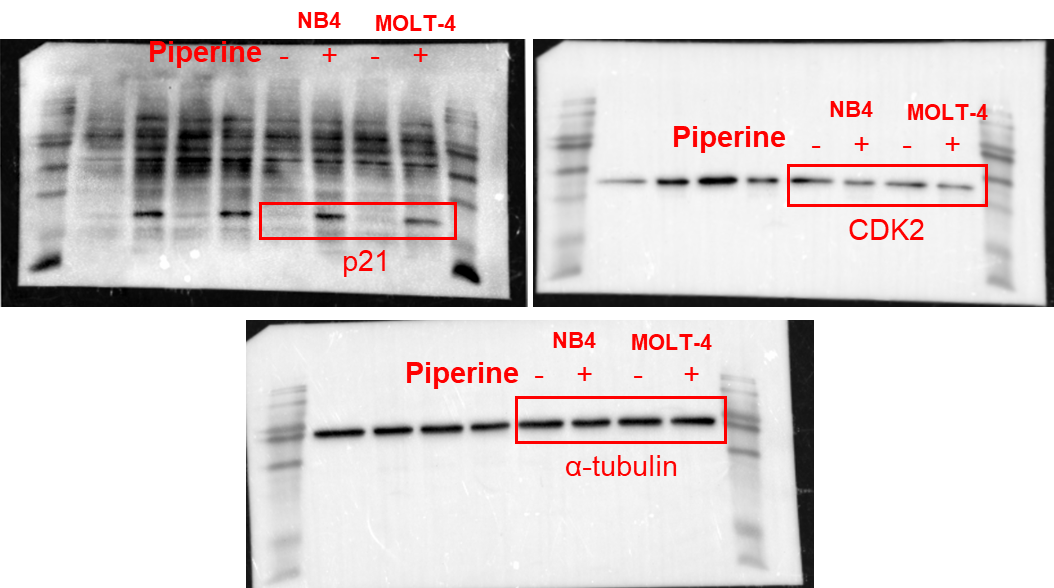
**

**Figure S2 All three replicate protein expression of western blot.** (A) The presence of mTOR, Beclin-1, ULK1 and α-tubulin in three replicates. Red squares were the proteins bands which were used to construct in a research article. Blue squares were used to analyze relative protein expressions. (B) The presence of p21, CDK2 and α-tubulin in three replicates. Red squares were the proteins band used in the Figure results. Both Red and Blue squares were used to analyze relative protein expressions.

**(A)**


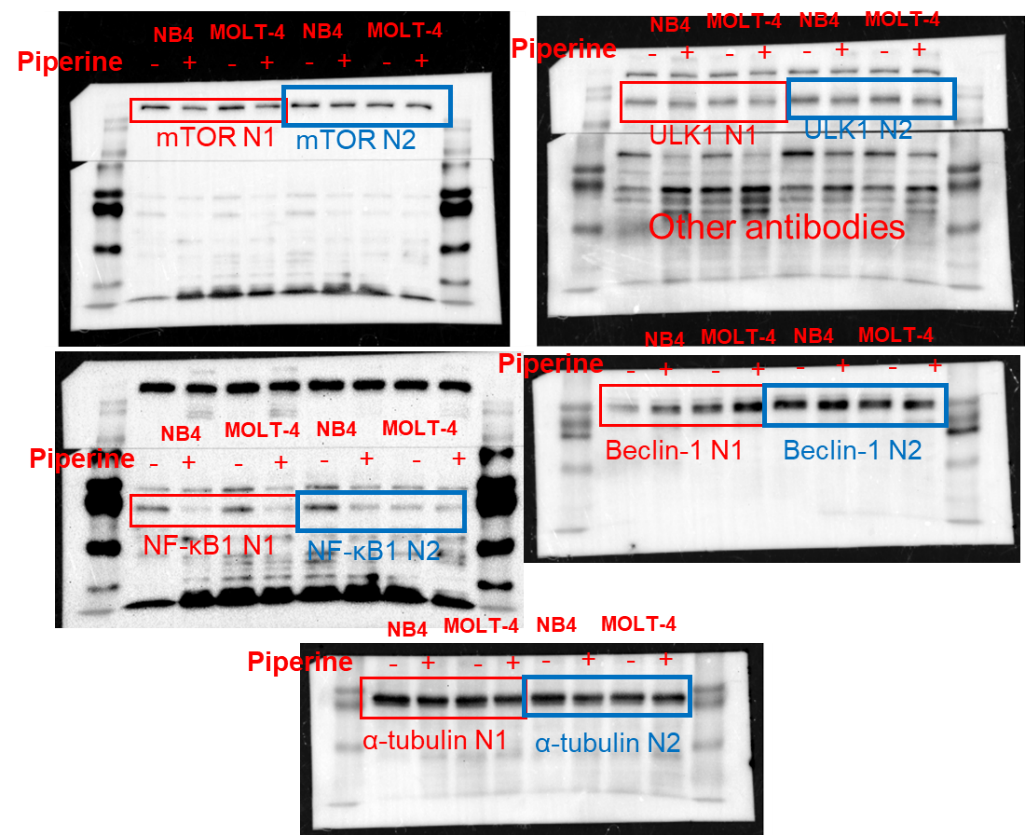


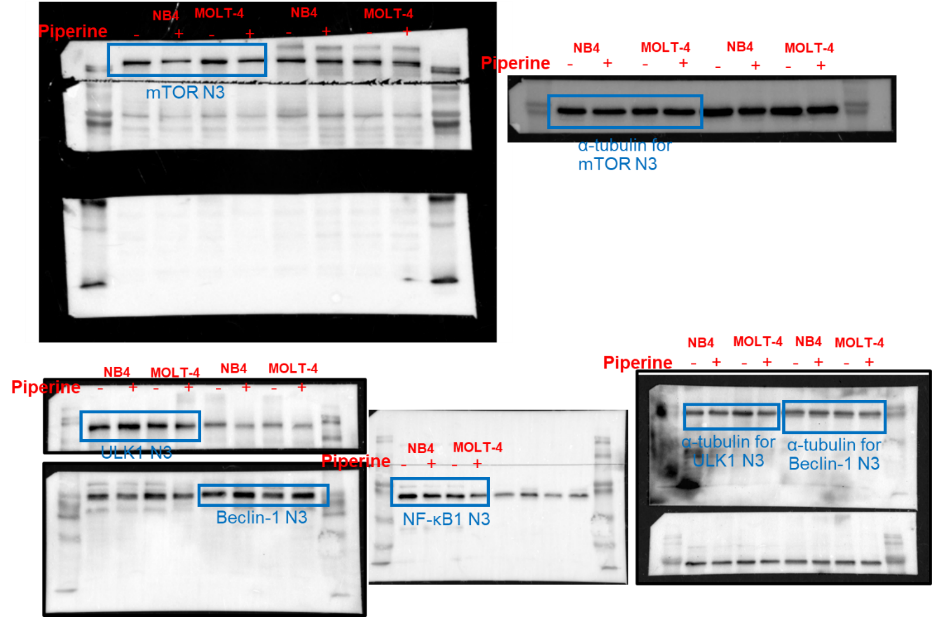


**(B)**


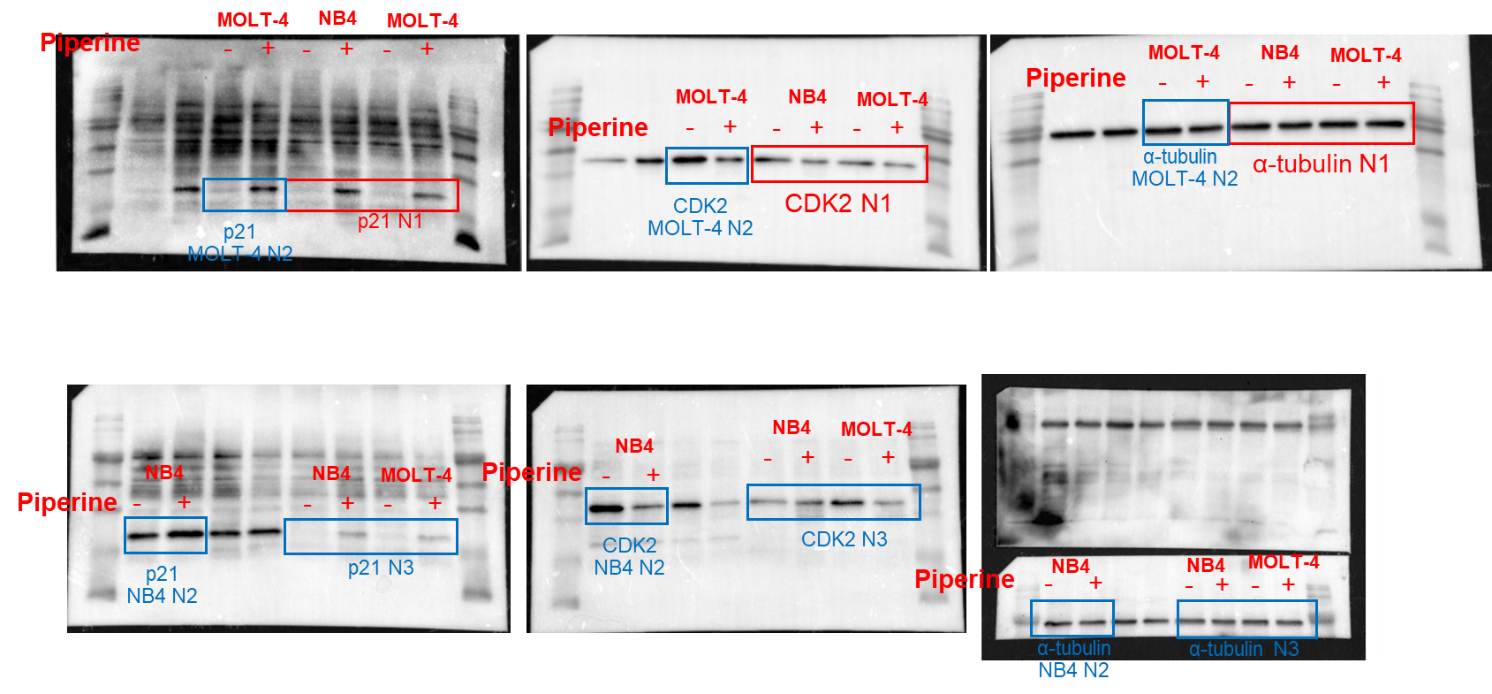

Supplement: Supplementary file 3 — Supplementary Material 3 [file 12906_2024_4641_MOESM3_ESM.docx]
